# Identification of QTNs, QTN-by-environment interactions and genes for yield-related traits in rice using 3VmrMLM

**DOI:** 10.3389/fpls.2022.995609

**Published:** 2022-10-17

**Authors:** Jin Zhang, Shengmeng Wang, Xinyi Wu, Le Han, Yuan Wang, Yangjun Wen

**Affiliations:** ^1^ College of Science, Nanjing Agricultural University, Nanjing, China; ^2^ Key Laboratory of Crop Genetics and Germplasm Enhancement, Nanjing Agricultural University, Nanjing, China

**Keywords:** 3VmrMLM, gwas, QTN-by-environment interaction, yield traits, rice

## Abstract

Rice, which supports more than half the population worldwide, is one of the most important food crops. Thus, potential yield-related quantitative trait nucleotides (QTNs) and QTN-by-environment interactions (QEIs) have been used to develop efficient rice breeding strategies. In this study, a compressed variance component mixed model, 3VmrMLM, in genome-wide association studies was used to detect QTNs for eight yield-related traits of 413 rice accessions with 44,000 single nucleotide polymorphisms. These traits include florets per panicle, panicle fertility, panicle length, panicle number per plant, plant height, primary panicle branch number, seed number per panicle, and flowering time. Meanwhile, QTNs and QEIs were identified for flowering times in three different environments and five subpopulations. In the detections, a total of 7~23 QTNs were detected for each trait, including the three single-environment flowering time traits. In the detection of QEIs for flowering time in the three environments, 21 QTNs and 13 QEIs were identified. In the five subpopulation analyses, 3~9 QTNs and 2~4 QEIs were detected for each subpopulation. Based on previous studies, we identified 87 known genes around the significant/suggested QTNs and QEIs, such as LOC_Os06g06750 (*OsMADS5*) and LOC_Os07g47330 (*FZP*). Further differential expression analysis and functional enrichment analysis identified 30 candidate genes. Of these candidate genes, 27 genes had high expression in specific tissues, and 19 of these 27 genes were homologous to known genes in *Arabidopsis*. Haplotype difference analysis revealed that LOC_Os04g53210 and LOC_Os07g42440 are possibly associated with yield, and LOC_Os04g53210 may be useful around a QEI for flowering time. These results provide insights for future breeding for high quality and yield in rice.

## Introduction

Rice (*Oryza sativa* L.), one of the most important food crops, supports more than half the population in the world. Therefore, rice is crucial to improving the safety, quality, stability, and sustainability of the global food supply (Muthayya et al., 2014). In China, rice production is second only to maize, accounting for 31.64% of the total grain produced in 2020 (http://www.stats.gov.cn/tjsj/ndsj/, accessed on June 2022). Moreover, from 1994 to 2020, rice accounted for 27.17% of the total grain produced in the world, which is 657.85 million tons per year (http://www.fao.org/faostat/en/#data/QC/visualize, accessed on June 2022). There is an urgent, ongoing global demand for highly productive rice varieties due to growth in the human population in particular in developing nations, in which rice is the primary source of calories (Toriyama, 2005); climate change; and the labor-, land-, and water-intensive nature of rice cultivation (Greenland, 1997). Furthermore, climate has an impact on the most crucial traits of rice, such as production and quality. Weather catastrophes are becoming increasingly severe across the world because of accelerating global climate change, which poses a significant challenge to the production of sustainable food. Developing resilient crops is an efficient strategy for coping with climate change. A wealth of plant breeding and genomic resources have been developed by the scientific community to assist in this endeavor, including high-quality genome sequences (Goff et al., 2002; Yu et al., 2002), dense SNP maps (McNally et al., 2009; Ebana et al., 2010; Huang et al., 2010), extensive germplasm collections (Ebana et al., 2008; McNally et al., 2009; Agrama et al., 2010), and public databases of genomic information (Tanaka et al., 2008; McNally et al., 2009; Huang et al., 2010; Youens-Clark et al., 2011). Yet despite the emergence of these scientific resources, traditional quantitative trait locus linkage mapping is most often used to understand the genetic structures of complex traits in rice.

Genome-wide association study (GWAS) mapping enables the simultaneous screening of huge numbers of accessions for genetic variation in a variety of complex traits. Humongous genetic variants for agronomic and economic traits have been extensively studied using single-locus GWAS methods, such as MLM (Zhang et al., 2005; Yu et al., 2006), EMMA (Kang et al., 2008), and GEMMA (Zhou and Stephens, 2012). Such single-locus GWAS methods have a limited ability in detecting quantitative trait nucleotides (QTNs) with marginal effects that are affected by the polygenic background and stringent Bonferroni correction (Wang et al., 2016). Even if adjusting for polygenic background enhances the statistical power of QTN detection, it is still difficult to identify the majority of small-effect QTNs related to complex traits using single-locus GWAS methods.

To address the issue in single-locus GWAS methods, multi-locus GWAS methods were developed as a multidimensional method of genome analysis, which simultaneously estimate the effects of all markers (Cui et al., 2018). In particular, to address the selection of cofactors in multi-locus GWAS models with millions of markers, researchers have proposed MLMM (Segura et al., 2012), FarmCPU (Liu et al., 2016), mrMLM (Wang et al., 2016), pLARmEB (Zhang et al., 2017), and FASTmrEMMA (Wen et al., 2018). However, the dominance (d) or QTN-by-environment interaction (QEI) were not fully considered in the above models. Moreover, when additive (a) and dominance (d) effects, additive-by-environment (a×e) interaction, dominance-by-environment (d×e) interaction, and their polygenic backgrounds are simultaneously included as random effects in a mixed model of genome-wide analysis, there are 10 variance components, which creates a huge computational burden.

To improve calculation efficiency, a mixed model with three variance components was combined with mrMLM to establish a new methodological framework, namely, 3VmrMLM, that identifies all types of loci and estimates their effects while controlling all possible polygenic backgrounds (Li et al., 2022a). In GWAS, QEI can be used extensively to explore the genetic structures of complex traits to meet the needs of phenotypic plasticity research and global climate change. 3VmrMLM was expanded to cover QEI using the same thinking as in QTN detection models.

The data set of 413 rice accessions with 44,000 SNPs from the Rice Diversity database (www.ricediversity.org, accessed on April 2022) is suitable for GWAS, which has been performed by many researchers. Although this data set contains a wealth of information, including data on yield-related traits closely related to human life, phenotypic data on a given trait in different locations, and data on different subpopulations with the same trait, it has been seldom studied for further both QTN and QEI detection simultaneously. Therefore, in this study, we reanalyzed eight yield-related traits in this natural population of 413 rice accessions using the proposed multi-locus method, 3VmrMLM. Our goals were to detect the significant QTNs and QEIs related to rice yield, mine candidate genes, speed up molecular marker-assisted breeding, and increase rice production.

## Material and methods

### Phenotypic data and statistical analysis

We used 3VmrMLM (Li et al., 2022a, 2022b) to reanalyze 413 accessions with 36,901 SNPs in rice (*Oryza sativa* L.) in Zhao et al. (2011) to detect significant QTNs and QEIs for eight yield-related traits. Phenotypic data were downloaded from the Rice Diversity database (www.ricediversity.org, accessed on April 2022). The yield-related agronomic traits were florets per panicle (FPP), panicle fertility (PF), panicle length (PL), panicle number per plant (PNPP), plant height (PH), primary panicle branch number (PPBN), seed number per panicle (SNPP), and flowering time in three environments, Aberdeen (FTAB), Arkansas (FTAR), and Faridpur (FTF). In Zhao et al. (2011), detailed information on the experimental designs is described. Flowering time at the three locations (FTAB, FTAR, and FTF) was used to detect QEIs for multi-environment analysis and also to detect QTNs for single-environment analysis. The other seven traits were phenotyped at the same locations for single-environment analysis to detect QTNs in this study.

To illustrate the variability of gene-environment interactions in subpopulations in rice, we also analyzed rice flowering time in FTAB, FTAR, and FTF for five subpopulations derived from Zhao et al. (2011), including Admixed (ADMIX), Australia (AUS), Indica (IND), Temperate japonica (TEJ), and Tropical japonica (TRJ), with sample sizes of 43, 50, 52, 69, and 78, respectively.

**Figure 1:**
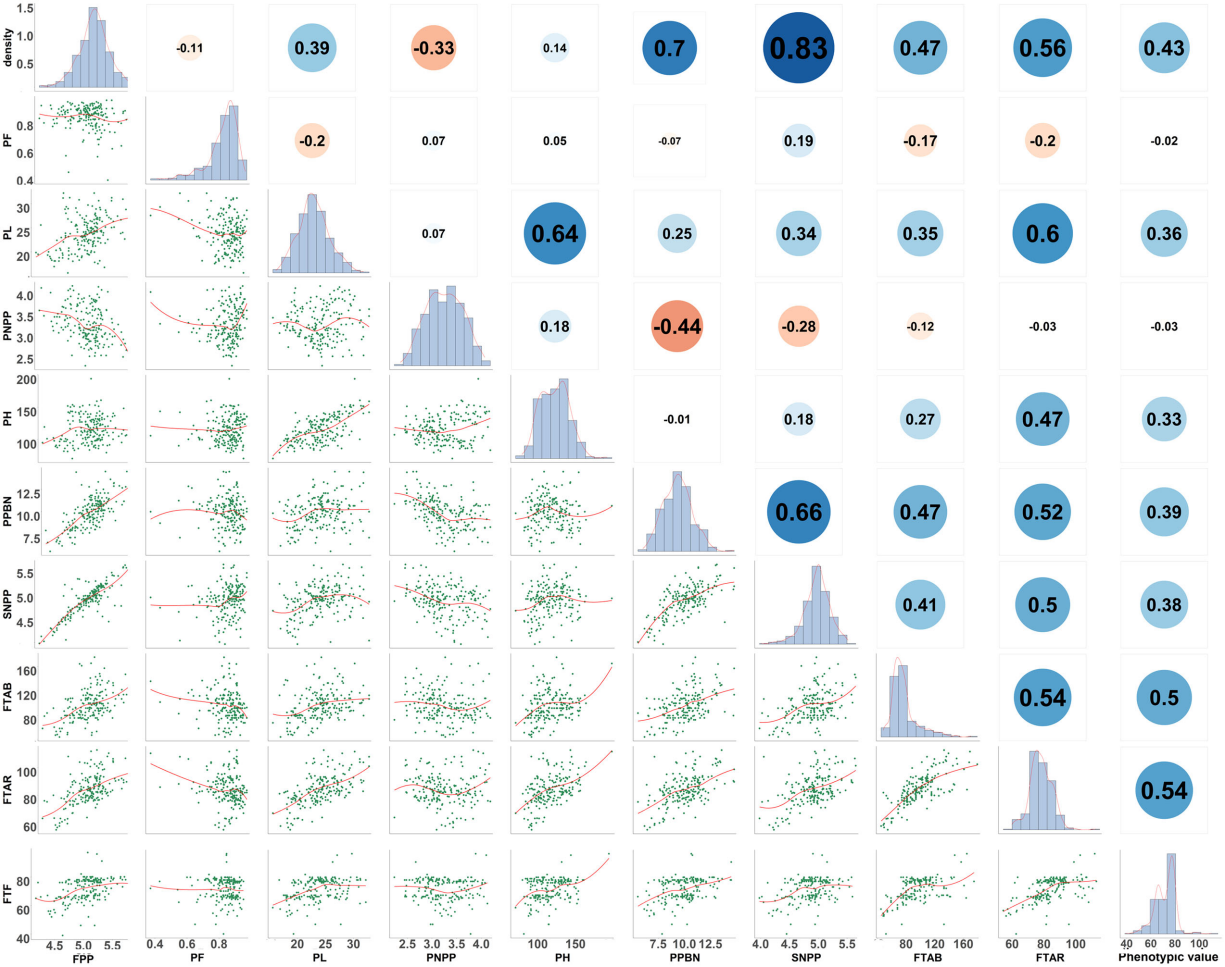
Distribution of eight yield-related traits in rice and Pearson coefficients. FTAB, FTAR, and FTF are the flowering time in three different environments in the single-environment analysis. Linear regression statistics between the two traits are below the diagonal, the diagonal histogram represents the distribution of each trait, and correlation coefficients are above the diagonal (positive numbers represent positive correlations, negative numbers represent negative correlations).

To visualize all eight traits, descriptive statistical analysis for each phenotypic data was performed, including the mean, minimum, maximum, range, standard deviation, and coefficient of variation (CV) for each trait (**Table 1**). Pearson correlation analysis (**Figure 1**) for all phenotypic data was performed in R version 4.1.2 (https://www.r-project.org/).

**Table 1:** Statistical analysis of eight rice yield-related traits.

Trait	Mean	Max	Min	SD	CV
FPP	5.056	5.836	3.909	0.323	0.064
PF	0.824	0.980	0.372	0.105	0.127
PL	24.375	35.683	15.633	3.537	0.145
PNPP	3.247	4.172	2.234	0.413	0.127
PH	116.583	194.333	67.750	21.092	0.181
PPBN	9.943	17.000	5.556	1.781	0.179
SNPP	4.854	5.635	3.445	0.330	0.068
FTAB^a^	107.050	306.000	45.000	38.957	0.364
FTAR^a^	87.944	150.500	54.500	12.627	0.144
FTF^a^	71.770	110.000	39.000	8.510	0.119

a indicates flowering time in three different environments in the single-environment analysis.

### Genotypic data

**Figure 2:**
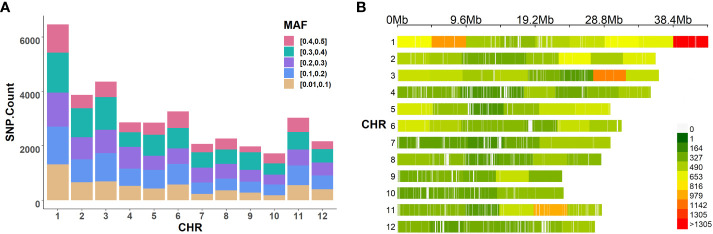
The distribution of SNPs in rice. **(A)** The distribution of minor allele frequency. **(B)** The density distribution of SNPs.

Genotypic data for the 413 rice accessions were obtained from the Rice Diversity database (www.ricediversity.org, accessed on April 2022). The data set consisted of a well-distributed 36,901 SNP array across the 12 chromosomes of rice with call rate > 70% and minor allele frequency > 0.01 (Zhao et al., 2011). To visualize the genotype in this study, **Figures 2A, B** illustrate the distribution of the minor allele frequency and the density distribution of loci on each chromosome. These were relatively uniform, which indicates that this data set is suitable for genetics dissection in rice.

### Gwas

The IIIVmrMLM software (Li et al., 2022b) of 3VmrMLM method (Li et al., 2022a) was downloaded from github (https://github.com/YuanmingZhang65/IIIVmrMLM). We performed QTN and QEI detection using the *IIIVmrMLM* function, specifying the parameters of “*=Single_env*” for the QTN detection model and “*=Multi_env*” for the QEI detection model. The thresholds of significant and suggested QTN or QEI were set at P-value = 0.05/*m* and LOD = 3.00, respectively, where *m* is the number of markers (Li et al., 2022a).

### SNP annotation and the identification of known genes

The China Rice Data Center database (https://ricedata.cn/, accessed on June 2022) was used to annotate the genes around significant/suggested QTNs and QEIs identified by 3VmrMLM. For all identified loci, regions within 200 kb were used to search for known genes (which were reported in previous studies and identified by 3VmrMLM simultaneously) according to linkage disequilibrium decay.

### Functional enrichment analysis and the identification of candidate genes

We performed differential expression analysis using the online tool GEO2R (https://www.ncbi.nlm.nih.gov/geo/geo2r/, accessed on September 2022) on four data sets (GSE19024, GSE21396, GSE136746, and GSE166053) from the Gene Expression Omnibus database (https://www.ncbi.nlm.nih.gov/geo/, accessed on September 2022). The datasets contain transcriptomic data related to rice development. Differentially expressed genes (DEGs) were screened by adjusted P-values less than 0.05, and then intersected with genes around significant/suggested QTNs or QEIs to obtain DEGs significantly associated with the target traits. For the functional annotation analysis, information of the above DEGs related to the target traits was submitted to the web-based tool DAVID (https://david.ncifcrf.gov/home.jsp, accessed on September 2022) to perform Kyoto Encyclopedia of Genes and Genomes functional enrichment analysis. Fisher’s exact test (P < 0.05) was used to select enrichment KEGG pathways. Genes that were enriched in these significant pathways were considered as candidate genes.

### Tissue specific expression and blast of homologous genes in *Arabidopsis*

The database Rice Genome Annotation Project (http://rice.uga.edu/, accessed on September 2022) was used to investigate the expression of all candidate genes in various tissues to further illustrate the association between genes and phenotypic variations. The R package *pheatmap* was used to create a heatmap of the FPKM expression of the candidate genes. Protein sequence information of the candidate genes was submitted to the Rice Genome Annotation Project (http://rice.uga.edu/analyses_search_blast.shtml, accessed on September 2022) to mine homologous *Arabidopsis* genes.

### Analysis of haplotype and phenotypic difference

To validate the associated loci between candidate genes and traits, the *HaploView* software package (http://www.broad.mit.edu/mpg/haploview/; Barrett et al., 2005) was used to perform linkage disequilibrium and haplotype block analyses and to estimate the frequency of haplotype populations in candidate genes. For each gene, significant variations were used for haplotype division, and the phenotypic differences between haplotypes was analyzed via *t* test using the *t.test* function in R.

## Results

### Phenotypic variation

Eight yield-related traits (including FPP, PF, PL, PNPP, PH, PPBN, SNPP, and flowering time in FTAB, FTAR, and FTF) were reanalyzed to determine whether there exists any significant genetic variation in these traits across 413 rice accessions. Descriptive statistics for all traits are listed in **Table 1**. Let us consider CV as an example, for flowering time in each single-environment, FTAB had the highest CV at 36.4%, which indicates that flowering time at Aberdeen had the largest variation. Furthermore, the CVs for FTAR and FTF were 14.4% and 11.9%, both relatively large, which indicates large variation and environmentally sensitive for flowering time. In addition, the CVs for the other six traits (PF, PL, PNPP, PH, PPBN, and SNPP) were 12.7%, 14.5%, 12.7%, 18.1%, 17.9%, and 6.8%, and FPP had the lowest CV at 6.4%.

Pearson correlation coefficients (PCCs) were calculated among the eight traits (**Figure 1**). FPP and PNPP were negatively correlated (PCC = –0.33), and a negative correlation was also observed between FPP and PF (PCC = –0.11). FPP was positively correlated with PPBN (PCC = 0.7) and SNPP (PCC = 0.83). In addition, PL was positively correlated with PH (PCC = 0.64), SNPP (PCC = 0.34), and FPP (PCC = 0.39), which indicates the close genetic relationship between panicle length and panicle number. With regard to flowering time across environments, FTAB was positively correlated with FTAR (PCC = 0.74) and FTF (PCC = 0.50), and FTAR and FTF were positively correlated (PCC = 0.54). These results demonstrate that the eight rice traits play a crucial role in controlling the rice yield and significantly correlate to one another.

### Identification of QTNs for yield-related traits using 3VmrMLM

We reanalyzed all eight yield-related traits using the single-environment QTN detection model in 3VmrMLM to identify QTNs, where flowering time was measured in three different environments. A total of 165 significant/suggested QTNs (**Supplementary Table S1**; **Supplementary Figure S1**) were detected as associated with at least one of the eight yield-related traits. Of these QTNs, 17, 16, 16, 21, 23, 17, 15, 15, 18, and 7 QTNs (**Supplementary Table S1**; **Supplementary Figure S1**) were associated with FPP, PF, PL, PNPP, PH, PPBN, SNPP, FTAB, FTAR, and FTF, respectively. The proportion of total phenotypic variance explained by QTNs for each single trait were 72.61%, 73.29%, 75.48%, 51.99%, 64.17%, 71.64%, 58.55%, 58.04%, 77.07%, and 44.60% calculated by the R package *IIIVmrMLM*. It shows that most QTNs had only additive effects. Note that some QTNs, such as id3005865 for FPP, id5014747 for PF, and id4007762 for PH, had both additive and dominance effects.

A total of 17 QTN hotspots (**Supplementary Table S1**; **Supplementary Figure S1A**) were detected as significantly associated with FPP, with P-values of 2.19E-32~7.60E-07 and LOD scores of 5.31~31.66, respectively. A total of 16 QTNs (**Supplementary Table S1**; **Supplementary Figure S1B**) associated with PF were detected with P-values of 1.08E-44~1.07E-06 and LOD scores of 4.97~32.90. A total of 16 QTNs (**Supplementary Table S1**; **Supplementary Figure S1C**) were associated with PL, with P-values of 2.33E-56~1.02E-05 and LOD scores of 4.23~54.34, and id7004886 located on chromosome 7 had the maximum phenotypic variance explained at 22.04% (**Supplementary Table S1**). Moreover, 21 QTNs (**Supplementary Table S1**; **Supplementary Figure S1D**) associated with PNPP were detected with P-values of 1.78E-37~9.71E-06. For PH, 23 QTNs (**Supplementary Table S1**; **Supplementary Figure S1E**) were detected with P-values of 1.15E-38~7.60E-05 and LOD scores of 3.40~37.94. A total of 18 QTNs (**Supplementary Table S1**; **Supplementary Figure S1F**) were detected as associated with PPBN; they were widely located on chromosomes 1, 2, 4, and 9, with P-values of 2.05E-39~1.31E-05 and LOD scores of 4.13~37.47. Among these QTNs, id1009181 located on chromosome 1 explained 16.03% of the phenotypic variance. For SNPP, 15 QTNs (**Supplementary Table S1**; **Supplementary Figure S1G**) were detected with P-values of 3.95E-41~2.11E-05 and LOD scores of 3.93~39.18. For the three flowering time environments, 30 QTNs (**Supplementary Table S1**; **Supplementary Figure S1H–J**) were detected on all chromosomes except chromosome 12 were detected, with P-values of 1.15E-32~2.17E-05 and LOD scores of 1.40~11.30. id4000121, ud7002024, and id4004217 explained the maximum phenotypic variance, which were 14.47%, 11.30%, and 7.75%, respectively.

### Known genes around significant/suggested QTNs

We compared genomic regions of 165 significant/suggested QTNs (200 kb up- and down-stream of each significant/suggested QTN) to the genomic positions of reported genes related to rice yield. A total of 73 known genes were around the significant/suggested QTNs, including 9, 7, 3, 14, 17, 6, 6, 2, 7, and 2 known genes for FPP, PF, PL, PNPP, PH, PPBN, SNPP, FTAB, FTAR, and FTF, respectively (**Table 2**; **Supplementary Figure S1**). Marker id1019150 located on chromosome 1 around LOC_Os01g54810 was simultaneously associated with PL and PH (**Table 2**; **Supplementary Figure S1**). Moreover, id1002863 and id7004587 around LOC_Os01g07480 and LOC_Os07g41250, respectively, on chromosomes 1 and 7 were associated with FPP and SNPP (**Table 2**; **Supplementary Figure S1**). It is interesting that a QTN can overlap with multiple known genes (e.g., three genes, LOC_Os02g45054, LOC_Os02g45070, and LOC_Os02g45110 were simultaneously around id2012042 on chromosome 2, **Table 2**; **Supplementary Figure S1**). *sd1* is associated with PH (Zhao et al., 2011). Moreover, *OsRA2*, located on chromosome 1 and simultaneously associated with FPP and SNPP, modifies panicle architecture by regulating pedicel length (Leran et al., 2014; Lu et al., 2017). *OsPTR4* controls FPP and SNPP (Leran et al., 2014).

**Table 2:** Known genes identified for rice yield-related traits using the QTN detection model in 3VmrMLM.

Trait	Marker	Chr	Position	add	dom	Variance	r^2^(%)	Gene Symbol	ID
FPP	id1002863	1	3481990	-0.049	–	0.002	1.700	*OsRA2*	LOC_Os01g07480
	id1002863	1	3481990	-0.049	–	0.002	1.700	*FIB*	LOC_Os01g07500
	id1003144	1	3801746	-0.057	–	0.003	2.030	*OsRE1*	LOC_Os01g07880
	id3000495	3	871080	0.117	–	0.007	5.714	*Ehd4^b^*	LOC_Os03g02160
	id7004587	7	24790535	0.056	–	0.002	1.338	*OsPTR4*	LOC_Os07g41250
	id7004587	7	24790535	0.056	–	0.002	1.338	*OsMADS18*	LOC_Os07g41370
	id7005660	7	28221129	-0.124	–	0.010	7.409	*OsCOL13*	LOC_Os07g47140
	id7005660	7	28221129	-0.124	–	0.010	7.409	*FZP*	LOC_Os07g47330
	id12009959	12	27218159	0.083	–	0.007	5.143	*OsPAP10c*	LOC_Os12g44020
PF	id1023500	1	37274860	-0.024	–	0.000	2.519	*OsABI5*	LOC_Os01g64000
	id1023500	1	37274860	-0.024	–	0.000	2.519	*REL1*	LOC_Os01g64380
	id3000828	3	1499569	-0.036	–	0.001	7.357	*OsmiR528*	LOC_Os03g03724
	id8007916	8	28208958	0.035	–	0.000	1.994	*OsNTL5*	LOC_Os08g44820
	id9002415	9	7894310	0.027	–	0.001	3.126	*OsEMF2b*	LOC_Os09g13630
	id9002415	9	7894310	0.027	–	0.001	3.126	*SDG724*	LOC_Os09g13740
	id12006848	12	21130413	0.046	–	0.000	2.8543	*OsVIL2*	LOC_Os12g34850
PL	id1017530	1	29565162	0.788	–	0.574	3.375	*OsLFL1*	LOC_Os01g51610
	id1019150	1	31662509	-1.275	–	1.134	6.672	*THIS1^b^*	LOC_Os01g54810
	id10003476	10	13216045	-1.350	–	1.544	9.088	*Brd2*	LOC_Os10g25780
PNPP	id1001128	1	1401052	-0.047	–	0.002	1.257	*MHZ4*	LOC_Os01g03750
	id2000516	2	647801	-0.086	–	0.003	1.662	*DHD4*	LOC_Os02g01990
	id2012042	2	27371812	-0.131	–	0.004	2.237	*SID1*	LOC_Os02g45054
	id2012042	2	27371812	-0.131	–	0.004	2.237	*OsAGO1a*	LOC_Os02g45070
	id2012042	2	27371812	-0.131	–	0.004	2.237	*OsMTA2*	LOC_Os02g45110
	id3003977	3	7327105	-0.088	–	0.008	4.398	*OsAPC6*	LOC_Os03g13370
	id3003977	3	7327105	-0.088	–	0.008	4.398	*LPA1*	LOC_Os03g13400
	id3006138	3	12008635	0.046	–	0.002	1.185	*OsPHR1*	LOC_Os03g21240
	id4010447	4	30843940	-0.062	–	0.004	2.125	*OsAP2-39*	LOC_Os04g52090
	id5011783	5	25197731	0.109	–	0.012	6.746	*OsmtSSB1*	LOC_Os05g43440
	id7000258	7	1588172	-0.079	–	0.005	2.844	*OSH15*	LOC_Os07g03770
	id8001120	8	3438707	0.050	–	0.002	1.427	*OsCOMT*	LOC_Os08g06100
	id8001120	8	3438707	0.050	–	0.002	1.427	*OsCCA1*	LOC_Os08g06110
	ud8000279	8	4363409	-0.071	–	0.005	2.710	*DTH8*	LOC_Os08g07740
PH	id1018978	1	31452220	-4.470	–	13.040	2.931	*OsCesA4*	LOC_Os01g54620
	id1024441	1	38537795	7.133	–	17.140	3.853	*sd1^b^*	LOC_Os01g66100
	id1018978	1	31452220	-4.470	–	13.040	2.931	*THIS1*	LOC_Os01g54810
	id1018978	1	31452220	-4.470	–	13.040	2.931	*OsVOZ1*	LOC_Os01g54930
	id1024441	1	38537795	7.133	–	17.140	3.853	*OsCrll3*	LOC_Os01g66590
	id4007762	4	23286717	-7.695	3.480	15.326	3.445	*TDD1*	LOC_Os04g38950
	id4007762	4	23286717	-7.695	3.480	15.326	3.445	*OsALDH10A5*	LOC_Os04g39020
	id4007762	4	23286717	-7.695	3.480	15.326	3.445	*d11*	LOC_Os04g39430
	id4010574	4	31138553	3.210	–	9.981	2.243	*OsAP2-39*	LOC_Os04g52090
	id4010574	4	31138553	3.210	–	9.981	2.243	*OsKS1*	LOC_Os04g52230
	id4010574	4	31138553	3.210	–	9.981	2.243	*FC1*	LOC_Os04g52280
	id6004564	6	7097190	-3.206	–	8.230	1.850	*YPD1*	LOC_Os06g13050
	wd6000736	6	10282460	-3.939	–	10.962	2.464	*OsNF-YB9*	LOC_Os06g17480
	id7005417	7	27547556	-2.096	–	4.341	0.976	*Fd-GOGAT1*	LOC_Os07g46460
	id8006905	8	24940725	5.109	–	17.476	3.928	*RCN11*	LOC_Os08g39380
	id8006905	8	24940725	5.109	–	17.476	3.928	*OsDOG*	LOC_Os08g39450
	id9007929	9	22920706	2.891	–	7.000	1.574	*OsDRP1E*	LOC_Os09g39960
PPBN	id1009181	1	13926463	-0.807	–	0.640	16.026	*IPI1*	LOC_Os01g24880
	id1014302	1	24275703	-0.447	–	0.174	4.361	*OsATG7*	LOC_Os01g42850
	id1022478	1	35621886	0.593	–	0.335	8.400	*LAX1*	LOC_Os01g61480
	id1022478	1	35621886	0.593	–	0.335	8.400	*OsBAG4*	LOC_Os01g61500
	id1024948	1	39308177	-0.460	–	0.122	3.050	*EG1*	LOC_Os01g67430
	id3005659	3	10842947	0.479	–	0.091	2.291	*SSD1*	LOC_Os03g19080
SNPP	id1002863	1	3481990	-0.049	–	0.002	1.894	*OsRA2*	LOC_Os01g07480
	id1013159	1	22950277	0.171	–	0.004	3.481	*LOG*	LOC_Os01g40630
	id3005721	3	10922512	0.087	–	0.003	2.449	*SDG718*	LOC_Os03g19480
	id3005721	3	10922512	0.087	–	0.003	2.449	*SRL2*	LOC_Os03g19520
	id6015132	6	26966327	0.061	–	0.004	3.275	*OsSPL10*	LOC_Os06g44860
	id7004587	7	24790535	0.074	–	0.003	2.665	*OsPTR4*	LOC_Os07g41250
FTAB^a^	id1027324	1	42152363	-10.893	–	27.056	1.783	*OsMLH1*	LOC_Os01g72880
	id6002745	6	3330294	9.162	–	80.673	5.316	*OsMADS5*	LOC_Os06g06750
FTAR^a^	id1021120	1	34082456	-3.468	–	11.006	5.050	*OsGCD1*	LOC_Os01g58750
	id3002064	3	3766414	-4.491	–	19.461	8.930	*DPW^b^*	LOC_Os03g07140
	id3002064	3	3766414	-4.491	–	19.461	8.930	*CYP704B2^b^*	LOC_Os03g07250
	id3002064	3	3766414	-4.491	–	19.461	8.930	*OsSUT1^b^*	LOC_Os03g07480
	ud7001067	7	15702110	4.474	–	17.692	8.119	*ORMDL*	LOC_Os07g26940
	id9006822	9	19210667	-2.851	-8.826	3.567	1.637	*OsDFR2A*	LOC_Os09g32025
	id11011548	11	28322308	3.318	–	2.462	1.130	*EDT1*	LOC_Os11g47330
FTF^a^	id4004217	4	14176927	2.677	–	5.804	7.747	*OsACOS12*	LOC_Os04g24530
	id6006288	6	10090472	1.900	–	3.609	4.329	*OsNF-YB9*	LOC_Os06g17480

“-” indicates no dominance effect for this QTN. ^a^indicates flowering time in three different environments in the single-environment analysis. ^b^indicates known gene which was detected by 3VmrMLM and EMMA simultaneously.

### Detection of QEIs for rice flowering time using 3VmrMLM

In the multi-environment analysis, flowering time at three locations (Aberdeen, Arkansas, and Faridpur) was reanalyzed using the QEI detection model in 3VmrMLM to identify QEIs. A total of 21 significant/suggested QTNs (**Table 3**; **Supplementary Figure S2A**) and 13 significant/suggested QEIs (**Table 4**; **Supplementary Figure S2B**) were simultaneously detected. Among them, id6006118 located on chromosome 6 had additive-by-environment interaction and dominance-by-environment interaction in all three environments.

**Table 3:** Significant/suggested QTNs for rice flowering time in three environments detected using the QTN-by-environment detection model in 3VmrMLM.

Marker	CHR	Positions	LOD	add	dom	Variance	r^2^(%)	P-value	Reported Gene	Reference
id1001009	1	1095730	9.492	2.180	–	4.486	1.147	3.810E-11	–	–
id1007272	1	9815262	19.756	-3.224	–	3.810	0.974	1.456E-21	–	–
id1008137	1	11376832	16.619	-2.991	–	8.260	2.112	2.169E-18	*-*	–
id1012744	1	22493100	11.948	2.765	–	7.583	1.939	1.192E-13	*SaF*	Xie et al., 2017
id1014639	1	24595570	9.958	-2.241	–	2.662	0.681	1.272E-11	*-*	–
ud3000099	3	1400496	24.223	-3.680	–	12.997	3.323	4.490E-26	*-*	–
id3004539	3	8656816	34.038	-4.261	–	14.612	3.736	5.814E-36	*OsSTRL2*	Zou et al., 2017
id3008283	3	16551139	4.319	1.506	–	2.092	0.535	8.203E-06	*-*	–
dd3001061	3	27836287	17.230	3.294	–	8.646	2.211	5.220E-19	*-*	–
id4001482	4	3628149	8.959	2.138	–	4.134	1.057	1.335E-10	*-*	–
id4005251	4	17893016	9.178	-2.435	–	5.598	1.431	7.982E-11	*-*	–
id5000013	5	44370	11.405	2.395	–	4.778	1.222	4.259E-13	*-*	–
id5008977	5	21268048	21.538	3.386	–	6.812	1.742	2.302E-23	*-*	–
id5012857	5	26783289	13.681	2.640	–	2.135	0.546	2.068E-15	*-*	–
id6002690	6	3289852	27.063	3.816	–	13.218	3.380	6.148E-29	*OsMADS5*	Arora et al., 2007; Zhu et al., 2022
id6005322	6	8185001	49.742	-5.502	–	8.540	2.184	9.554E-52	*-*	–
ud7000660	7	8553942	15.086	-2.944	–	6.661	1.703	7.754E-17	*-*	–
id7004583	7	24784697	24.039	3.582	–	7.380	1.887	6.889E-26	*OsUAM3*	Konishi et al., 2007
id8000022	8	51045	23.711	-3.509	–	7.457	1.907	1.475E-25	–	–
id10000202	10	1012769	32.396	-4.148	–	12.160	3.109	2.618E-34	–	–
id110107061	11	26711260	13.869	-2.654	–	2.831	0.724	1.332E-15	–	–

“-” indicates no dominance effect or reported gene for this QTN.

**Table 4:** Significant/suggested QEIs for rice flowering time in three environments detected using the QTN-by-environment detection model in 3VmrMLM.

Marker	CHR	Positions	LOD	add1	dom1	add2	dom2	add3	dom3	Variance	r^2^(%)	P-value	Reported Gene	Reference
id1000015	1	149005	21.318	4.569	–	-1.336	–	-3.233	–	11.037	2.822	4.819E-22	–	–
id1000947	1	1042817	10.498	-3.007	–	0.422	–	2.586	–	5.303	1.356	3.180E-11	–	–
id1008137	1	11376832	14.763	-3.871	–	1.145	–	2.726	–	7.910	2.023	1.729E-15	–	–
ud2000978	2	17730153	9.539	-3.236	–	1.034	–	2.202	–	5.465	1.397	2.893E-10	–	–
id4002940	4	8211710	16.440	-4.001	–	1.251	–	2.749	–	8.377	2.142	3.637E-17	–	–
id5000766	5	1128994	10.402	-2.418	–	-0.647	–	3.064	–	5.218	1.334	3.971E-11	–	–
id6002690	6	3289852	6.107	1.782	–	0.622	–	-2.404	–	3.113	0.796	7.813E-07	*OsMADS5*	Arora et al., 2007; Zhu et al., 2022
id6005330	6	8234981	8.562	-2.930	–	1.146	–	1.785	–	4.362	1.115	2.747E-09	–	–
id6006118	6	9651785	33.572	-5.915	-0.010	2.086	1.406	3.829	-1.396	17.945	4.588	2.106E-32	–	–
id6007539	6	12322330	20.565	4.618	–	-1.690	–	-2.928	–	10.917	2.791	2.730E-21	–	–
id7004142	7	23351238	8.336	2.802	–	-0.792	–	-2.010	–	4.174	1.067	4.615E-09	–	–
id10006353	10	20022516	13.901	4.031	–	-1.259	–	-2.772	–	8.506	2.175	1.259E-14	–	–
id11006398	11	17823963	15.862	-3.984	–	1.500	–	2.484	–	8.097	2.070	1.377E-16	–	–

“-” indicates no dominance effect or reported gene for this QEI.

We compared genomic regions of the significant/suggested QTNs or QEIs (200 kb up- and down-stream around the significant/suggested QTNs or QEIs) to the positions of previously reported genes related to rice flowering time. 4 QTNs (**Table 3**; **Supplementary Figure S2A**) and 1 QEI (**Table 4**; **Supplementary Figure S2B**) overlapped with the known genes. Notably, id6002690, which was adjacent to LOC_Os06g06750 (*OsMADS5*), was demonstrated to have both QTN and QEI effects. Microarray-based expression profiling and genome-wide molecular characterization of the genes that encode the MADS-box transcription factor family was presented by Arora et al. (2007). *OsMADS5* in this gene family is associated with the development of inflorescence. Recently, Zhu et al. (2022) also revealed the function of *OsMADS5* in the development of inflorescence and showed that *OsMADS5* is involved in limiting branching and promoting the transition to spikelet meristem identity, partly by repressing RCN4 expression.

For five different subpopulations (ADMIX, AUS, IND, TEJ, and TRJ), flowering time in FTAB, FTAR, and FTF was also analyzed to illustrate the variability in gene-environment interactions. A total of 25 QTNs and 15 QEIs (**Supplementary Table S2**; **Supplementary Figures S2C–L**) were simultaneously detected with the multi-environment detection model in 3VmrMLM, including 3, 3, 6, 9, and 4 QTNs and 4, 2, 4, 3, and 2 QEIs for ADMIX, AUS, IND, TEJ, and TRJ, respectively. Note that there was no overlap in QEI between different subpopulations, which may indicate that these QEIs come from different ecological adaptations.

### Functional enrichment analysis of candidate genes

In addition to the aforementioned significant/suggested QTNs and QEIs with known genes, we also detected several new QTNs and QEIs that have not been reported in previous studies, such as id2005901, id6007721, id12008098, and id9001769 (**Supplementary Table S1**; **Supplementary Figure S1**). To identify the candidate genes, we considered genes in regions 200 kb up- and down-stream around each significant/suggested QTN and QEI, including all studies of population and each subpopulation. There are about 8000 genes within these 200kb regions, of which 755 are DEGs that show different expression between test and control groups of rice accessions.

**Figure 3:**
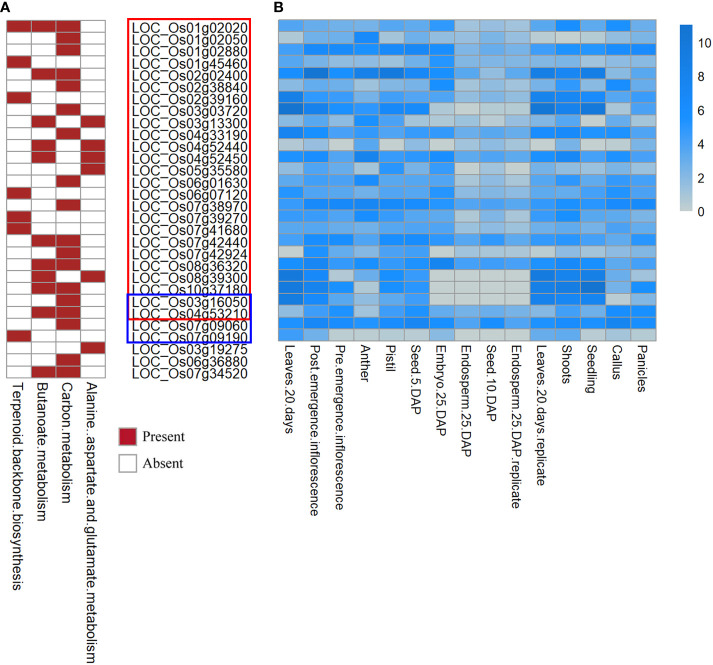
Heatmap of the functional enrichment analysis and tissue-specific expression analysis. **(A)** Heatmap of the functional enrichment analysis for the candidate genes. **(B)** Heatmap of FPKM expression for the part of candidate genes. The y-axis is log2(FPKM+1). Candidate genes in the red box correspond to QTNs. Blue box: QEIs for flowering time, remaining: candidate genes not expressed in specific tissue.

In the Kyoto Encyclopedia of Genes and Genomes analysis, 30 genes significantly involved in 4 biological processes (terpenoid backbone biosynthesis, butanoate metabolism, carbon metabolism, and alanine, aspartate and glutamate metabolism) were defined as candidate genes. **Figure 3A** shows results for the candidate genes in the rectangular boxes, the most significant pathways are marked in red.

The results of the functional enrichment analysis (**Figure 3A**) showed that some candidate genes around the new QTNs and QEIs were involved in many biological and metabolic processes during rice growth, which have not been reported in previous studies, such as flower development, which indicates that these candidate genes have a non-negligible influence on the target traits. For example, LOC_Os01g02020 (**Figure 3A**), a candidate gene detected in PH and SNPP, was involved in terpenoid backbone biosynthesis, butanoate metabolism, and carbon metabolism. In addition, the candidate gene LOC_Os04g52450 (**Figure 3A**) was directly involved in butanoate metabolism and in alanine, aspartate, and glutamate metabolism. Moreover, some candidate genes detected in the multi-environment analysis for each subpopulation, including LOC_Os03g16050 for IND, LOC_Os04g53210 for AUS, and LOC_Os07g09060 and LOC_Os07g09190 for TRJ (**Figure 3A**), were involved in a series of biological and metabolic processes.

### Expression profile of candidate genes

The Rice Genome Annotation Project database (http://rice.uga.edu) demonstrates the expression of the candidate genes in various tissues or organs, including shoots, roots, seeds, leaves, panicles, anthers, pistils, post-emergence, pre-emergence, and embryos. The heatmap of the candidate genes presented in **Figure 3B** shows the FPKM expression of the candidate genes in tissues and organs.

For QTN, LOC_Os04g52450 and LOC_Os08g36320 had high expression in leaves, panicles, shoots, and seedlings in rice (**Figure 3B**). Furthermore, LOC_Os03g16050 had the highest expression in pre-emergence inflorescence, leaves, shoots, and seedlings. Some earlier studies (Zhao et al., 2011; Weng et al., 2014) suggested that inflorescence, anthers, pistils, and panicles play important roles in regulating yield.

For QEIs of flowering time, LOC_Os03g16050, LOC_Os04g53210, LOC_Os07g09060 had high expression in post-emergence inflorescence and pre-emergence inflorescence, which might indicate a potential association between these candidate genes and flowering time (**Figure 3B**).

Among the 30 candidate genes, LOC_Os03g19275, LOC_Os06g36880, and LOC_Os07g34520 were not expressed in panicles or inflorescence; thus, these genes were not considered in further analyses. Among the 27 candidate genes identified here after tissue-specific expression analysis, 19 candidate genes are listed in **Table 5** for their homologous *Arabidopsis* genes.

**Table 5:** Orthologous information of candidate genes with higher tissue expression.

Trait	gene	Marker	*Arabidopsis* Orthologous gene	Putative function
FT_Q/TRJ_Q	LOC_Os01g02880	id1001009/id1001003	*AT2G01140*	Aldolase superfamily protein
TEJ_Q	LOC_Os01g45460	id1015276	*AT1G26120/AT3G02410/AT5G15860*	alpha/beta-Hydrolases superfamily protein/prenylcysteine methylesterase
PPBN	LOC_Os02g38840	id2009400	*AT3G27300/AT5G40760*	glucose-6-phosphate dehydrogenase 6
PPBN	LOC_Os02g39160	id2009400	*AT5G60600*	4-hydroxy-3-methylbut-2-enyl diphosphate synthase
PNPP	LOC_Os03g13300	id3003977	*AT5G17330*	glutamate decarboxylase
IND_QE/FT_Q	LOC_Os03g16050	id3004734/id3004539	*AT3G54050*	high cyclic electron flow 1
PF	LOC_Os04g33190	id4006172	*AT5G36880*	acetyl-CoA synthetase
AUS_QE/FTAB/FTF	LOC_Os04g53210	id4010914/id4010930/id4010984	*AT4G18360*	Aldolase-type TIM barrel family protein
FT_Q	LOC_Os05g35580	id5008977	*AT2G16570/AT4G34740*	GLN phosphoribosyl pyrophosphate amidotransferase 1/GLN phosphoribosyl pyrophosphate amidotransferase 2
PL/PH	LOC_Os06g01630	id6000302	*AT1G54220/AT3G13930*	Dihydrolipoamide acetyltransferase, long form protein
FTAB/FT_Q	LOC_Os06g07120	id6002745/id6002690	*AT2G17570*	Undecaprenyl pyrophosphate synthetase family protein
TRJ_QE	LOC_Os07g09060	id7000656	*AT2G14170*	aldehyde dehydrogenase 6B2
FT_QE	LOC_Os07g38970	id7004142	*AT5G08300/AT5G23250*	Succinyl-CoA ligase, alpha subunit
FT_QE	LOC_Os07g39270	id7004142	*AT2G18620/AT4G36810*	Terpenoid synthases superfamily protein/ geranylgeranyl pyrophosphate synthase 1
FT_Q/FPP/SNPP	LOC_Os07g41680	id7004583/id7004587	*AT2G17570*	Undecaprenyl pyrophosphate synthetase family protein
PH	LOC_Os07g42440	id7004779	*AT3G14130/AT3G14150*	Aldolase-type TIM barrel family protein
PL/FPP	LOC_Os07g42924	id7004886/id7004865	*AT1G22430/AT1G22440/AT4G22110*	GroES-like zinc-binding dehydrogenase family protein/Zinc-binding alcohol dehydrogenase family protein/GroES-like zinc-binding dehydrogenase family protein
PH	LOC_Os08g39300	id8006905	*AT2G13360*	alanine: glyoxylate aminotransferase
FT_QE	LOC_Os10g37180	id10006353	*AT1G32470/AT2G35370*	Single hybrid motif superfamily protein/ glycine decarboxylase complex H

Q and QE indicate significant/suggested QTNs and QEIs in the multi-environment analysis, respectively. AUS, IND, TEJ, and TRJ indicate subpopulations of the 413 rice accessions. Other abbreviations indicate results of the single-environment analysis.

### Haplotype and phenotypic difference analysis of candidate genes

**Figure 4:**
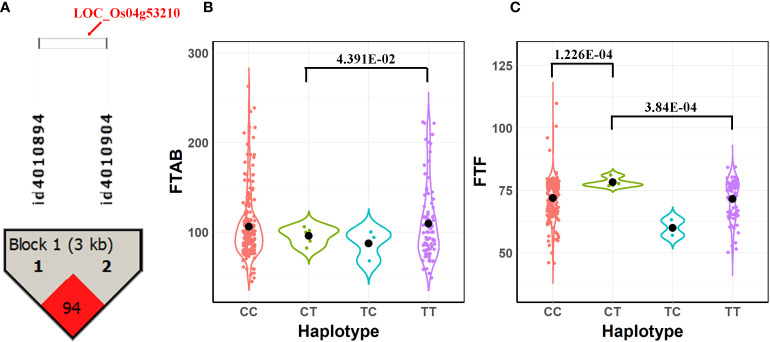
Results of haplotype and phenotypic difference analysis for the candidate gene LOC_Os04g53210. **(A)** Linkage disequilibrium and haplotype block with two SNPs inside for LOC_Os04g53210. **(B)** Comparison of FTAB among haplotypes CT, CC, TC, and TT. **(C)** Comparison of FTF among haplotypes CT, CC, TC, and TT.

To further verify the association between the candidate genes and target traits, we performed haplotype analysis of the candidate genes using SNPs within the candidate genes and 2 kb upstream of the candidate genes. LOC_Os04g53210 (CDS coordinates [5′-3′]: 31688717 ~ 31692592) was analyzed to reveal the intragenic variation affecting the rice yield and to identify favorable haplotypes. **Figure 4A** shows the linkage disequilibrium and haplotype block with two SNPs (id4010894 at 31688182 bp and id4010904 at 31691252 bp). The 413 accessions were classified into 4 haplotypes based on these two SNPs (id4010894 and id4010904). Among these haplotypes, haplotypes TT and CT had the highest mean phenotypic values of FTAB (109.54) and FTF (78.25), respectively, whereas haplotype TC presented the lowest FTAB (87.33) and FTF (60.00; **Figures 4B, C**). A *t* test showed that significant differences in FTAB and FTF existed between haplotypes CT and TT (P-values = 4.93E-02 and 3.84E-04, respectively). There was also a significant difference in FTF between haplotypes CT and CC (P-values = 1.23E-04). Therefore, we infer the candidate gene LOC_Os04g53210 to be associated with flowering in rice.

LOC_Os04g53210 was also detected in the multi-environment analysis for the AUS subpopulation. **Supplementary Figure S3A** shows the differences in phenotype among the 4 haplotypes. **Supplementary Figure S3B** shows the results of the haplotype block and phenotype difference in LOC_Os07g42440, which was detected in PH. We infer that the candidate gene LOC_Os04g53210 might be a gene-environment interaction for flowering time and that LOC_Os07g42440 might be associated with yield in rice.

## Discussion

Classic single-locus methods, such as MLM and general linear model (GLM), have been used extensively to detect genetic variants in many cereals (Price et al., 2006; Sant’Ana et al., 2018; He et al., 2019). However, these models suffer from multiple test corrections (e.g., Bonferroni correction) for critical values and neglect the overall effects of multiple loci (Zhong et al., 2021). For example, many robust quantitative trait loci, in particular small-effect quantitative trait loci, are missing because of the stringent threshold (Zhang et al., 2005). Therefore, multi-locus GWAS models, which are relatively closer to the real genetic architecture of animals and plants, have been developed. Geneticists developed these models to reduce the bias associated with estimating effects by controlling the population structure and polygenic background (Zhang et al., 2005; Yu et al., 2006; Zhang et al., 2010). In this study, a multi-locus GWAS method 3VmrMLM was used to detect QTNs for eight yield-related traits in 413 rice varieties with 36,901 SNPs. We detected 17, 16, 16, 21, 23, 17, 15, 15, 18, and 7 significant/suggested SNPs and 9, 7, 3, 14, 17, 6, 6, 2, 7, and 2 known genes for FPP, PF, PL, PNPP, PH, PPBN, SNPP, FTAB, FTAR, and FTF, respectively, using the QTN detection model in 3VmrMLM (**Supplementary Table S1**). Furthermore, we compared 3VmrMLM to a single-locus method, EMMA (Kang et al., 2008) by Zhao et al. (2011). We detected 4, 3, 3, 6, 5, 2, 1, 14, 6, and 2 QTNs by EMMA; thus, 3VmrMLM detected more significant QTNs than EMMA. Among these significant QTNs, 1, 1, 1, 1, 1, 0, 0, 1, 1, and 0 were detected by the two methods simultaneously, including id3000495, id2004552, id1019150, id12008894, id1101154, id8006573, and id3002064. 1, 0, 2, 1, 1, 0, 0, 1, 3, and 1 known gene were detected by EMMA, which were less than 3VmrMLM. Among these known genes, 6 were detected by EMMA and 3VmrMLM simultaneously, including *End4*, *TH1S1*, *sd1*, *DPW*, *CYP704B2*, and *OsSUT1* (**Table 2**). In addition to these 6 known genes, we identified 3 candidate genes for EMMA by performing differential expression analysis and functional enrichment analysis, and there was no overlap in candidate genes between the two methods. Moreover, the QTNs detected by 3VmrMLM explained a higher proportion of total phenotypic variance (72.61%, 73.29%, 75.48%, 51.99%, 64.17%, 71.64%, 58.55%, 77.07%, and 44.60%) than those detected by EMMA (17.1%, 8.1%, 10.9%, 7%, 38.6%, 6%, 0.1%, 31.3%, and 8.1%), except for FTAB. Overall, the multi-locus GWAS method are flexible to detect more QTNs and validate more known genes and candidate genes than the single-locus GWAS method.

The contribution of QEI to the genetic analysis of complex traits in plant, animal, and human genetics is growing. As a result of accelerating global climate change, weather disasters in a variety of regions are becoming increasingly severe, posing a substantial obstacle to sustainable food production. An efficient way of adapting to climate change is to develop climate-resilient crops. However, it is first necessary to detect QEIs and mine their genes. In addition, the environment has an impact on important traits, such as quality, yield, adaptability, and resistance, but studies on physiological effects, molecular mechanisms, and functional analyses of QEI genes under a variety of environments are not insightful enough because of the algorithms used. Moreover, joint analysis of multiple environments can enhance statistical power and experimental accuracy in the detection of QTN and QEI. In this study, three flowering time environments were used to identify QEIs for rice using a multi-environment detection model in 3VmrMLM, and 21, 3, 3, 6, 9, and 4 QTNs and 13, 4, 2, 4, 3, and 2 QEIs were detected for all populations and each subpopulation (**Tables 3**, **4**; **Supplementary Table S2**).

Pleiotropy was verified in this study. Among all the 165 significant/suggested QTNs for the eight traits detected using the QTN detection model in 3VmrMLM, some QTNs were significantly associated with more than one trait. 5 QTNs simultaneously related to FPP and SNPP were detected because of the strong correlation (PCC = 0.83) between these two traits, including id1002863, id3000495, id6009226, id7004587, and id11010822. Around these 5 QTNs, genes the *OsRA2*, *Ehd4*, and *OsPTR4* genes were identified (Gao et al., 2013; Lu et al., 2017; Huang et al., 2019). Id2005901 located on chromosome 2 was associated with both FPP and PPBN (PCC = 0.70). For PH and PL with a positive correlation (PCC = 0.64), id6000302 located on chromosome 6 was simultaneously detected. Moreover, id11011548 located on chromosome 11 was found to affect both PH and FTAR (PCC = 0.47), where the *EDT1* gene was identified (Bai et al., 2019).

Among the total of 117 genes around the significant/suggested QTNs and QEIs in this study, 87 were known genes that have been reported in previous studies. For these known genes with QTN effects (**Table 2**), *sd1*is associated with PH (Zhao et al., 2011). *OsMADS18* from the MADS-box transcription factor family affects panicle development (Kobayashi et al., 2012). Moreover, *OsRA2*, located on chromosome 1, which simultaneously affects FPP and SNPP, modifies panicle architecture by regulating pedicel length (Lu et al., 2017). Notably, *OsMADS5* was demonstrated to have both QTN effect and QEI effect, which was associated with inflorescence development in several previous studies (Arora et al., 2007; Zhu et al., 2022).

In addition to the above-mentioned 87 known genes, 30 candidate genes around the significant/suggested QTNs and QEIs that have not previously been reported were also detected in this study. These candidate genes were shown to be involved in many biological processes of rice growth, which indicates underlying associations between the identified candidate genes and the target traits (**Figure 3A**). Among these 30 candidate genes, 27 candidate genes had high expression in specific tissues, such as panicles and inflorescence (**Figure 3B**). In addition, 19 candidate genes associated with different traits had homologous genes in *Arabidopsis* (**Table 5**). LOC_Os04g53210 and LOC_Os07g42440 were demonstrated to be potentially associated with flowering and yield, respectively, by haplotype and phenotypic difference analysis (**Figure 4**; **Supplementary Figure S3B**). LOC_Os04g53210 especially might be a key gene in gene-environment interaction for flowering time (**Supplementary Figure S3A**).

3VmrMLM represents a significant advancement in GWAS methodologies and practical applications. First, 3VmrMLM correctly detects both QTNs and QEIs and produces unbiased estimations of their effects, unlike current GWAS methods that only detect QTNs and estimate genetic effects (Li et al., 2022a). Second, despite the fact that Feldmann et al. (2021) discovered that the phenotypic variance explained and the percentage of marker-associated genetic variance of large-effect loci were overestimated in analyses of complex traits, maximum likelihood estimation using ANOVA with the linear invariance property theoretically guarantees accurate loci detection and unbiased estimation of effects. Moreover, 3VmrMLM uses a compressed mixed model with three variance components to overcome the huge computational burden in traditional GWAS models. Therefore, 3VmrMLM is a good choice for detecting QTNs and QEIs associated with rice yield-related traits.

## Conclusion

In this study, a compressed mixed model with three variance components in GWAS, 3VmrMLM, was used to detect QTNs and QEIs related to rice yield traits. A total of 165 QTNs were identified. Moreover, 75 known genes were identified adjacent to the QTNs based on genome annotation and previous studies. In terms of QTN-by-environment detection, 21, 3, 3, 6, 9, and 4 QTNs and 13, 4, 2, 4, 3, and 2 QEIs were detected for all populations and each subpopulation. Moreover, 12 known genes were identified adjacent to the QTNs and QEIs. As a result of further differential expression and functional enrichment analysis, 30 candidate genes were detected. LOC_Os04g53210 and LOC_Os07g42440 were confirmed as main candidate genes by tissue-specific expression analysis, comparison of homologous *Arabidopsis* genes, and haplotype and phenotypic difference analysis. LOC_Os04g53210 might be useful in gene-environment interaction for a flowering time trait. These results could be helpful for detecting genes related to rice yield.

## Data availability statement

The datasets presented in this study can be found in online repositories. The names of the repository/repositories and accession number(s) can be found in the article/**Supplementary Material**.

## Author contributions

JZ and SW drafted the manuscript. JZ, SW, XW, LH, and YW analyzed the data. YJW and JZ conceived the study and were in charge of direction and planning. All authors contributed to the article and approved the submitted version.

## Funding

This research was supported by grants from the National Natural Science Foundation of China (32070688, 32270694, 31701071), the Postdoctoral Science Foundation of Jiangsu (2020Z330), and the Fundamental Research Funds for the Central Universities (JCQY202108).

## Conflict of interest

The authors declare that the research was conducted in the absence of any commercial or financial relationships that could be construed as a potential conflict of interest.

## Publisher’s note

All claims expressed in this article are solely those of the authors and do not necessarily represent those of their affiliated organizations, or those of the publisher, the editors and the reviewers. Any product that may be evaluated in this article, or claim that may be made by its manufacturer, is not guaranteed or endorsed by the publisher.

## Supplementary material

Supplementary Figure 1 Manhattan plots of the single-environment analysis for eight yield-related traits of rice. FTAB, FTAR, and FTF are the flowering time in three different environments. Pink text: known genes for the corresponding significant/suggested SNPs.

Supplementary Figure 2 Manhattan plots of the multi-environment analysis for the flowering time of rice. **(A, B)** Manhattan plots of QTNs and QTN-by-environment interactions for all populations. **(C–L)** Manhattan plots of QTNs and QTN-by-environment interactions for each subpopulation. Pink text: known genes for the corresponding significant/suggested SNPs.

Supplementary Figure 3 Results of haplotype and phenotypic difference analysis for the candidate genes. **(A)** LOC_Os04g53210. **(B)** LOC_Os07g42440.
