# Supplementary figures and images for: Identification of QTNs, QTN-by-environment interactions and genes for yield-related traits in rice using 3VmrMLM
Source: Front Plant Sci. 2022 Oct 17;13:995609. doi: 10.3389/fpls.2022.995609 (PMC9618716; doi:10.3389/fpls.2022.995609)

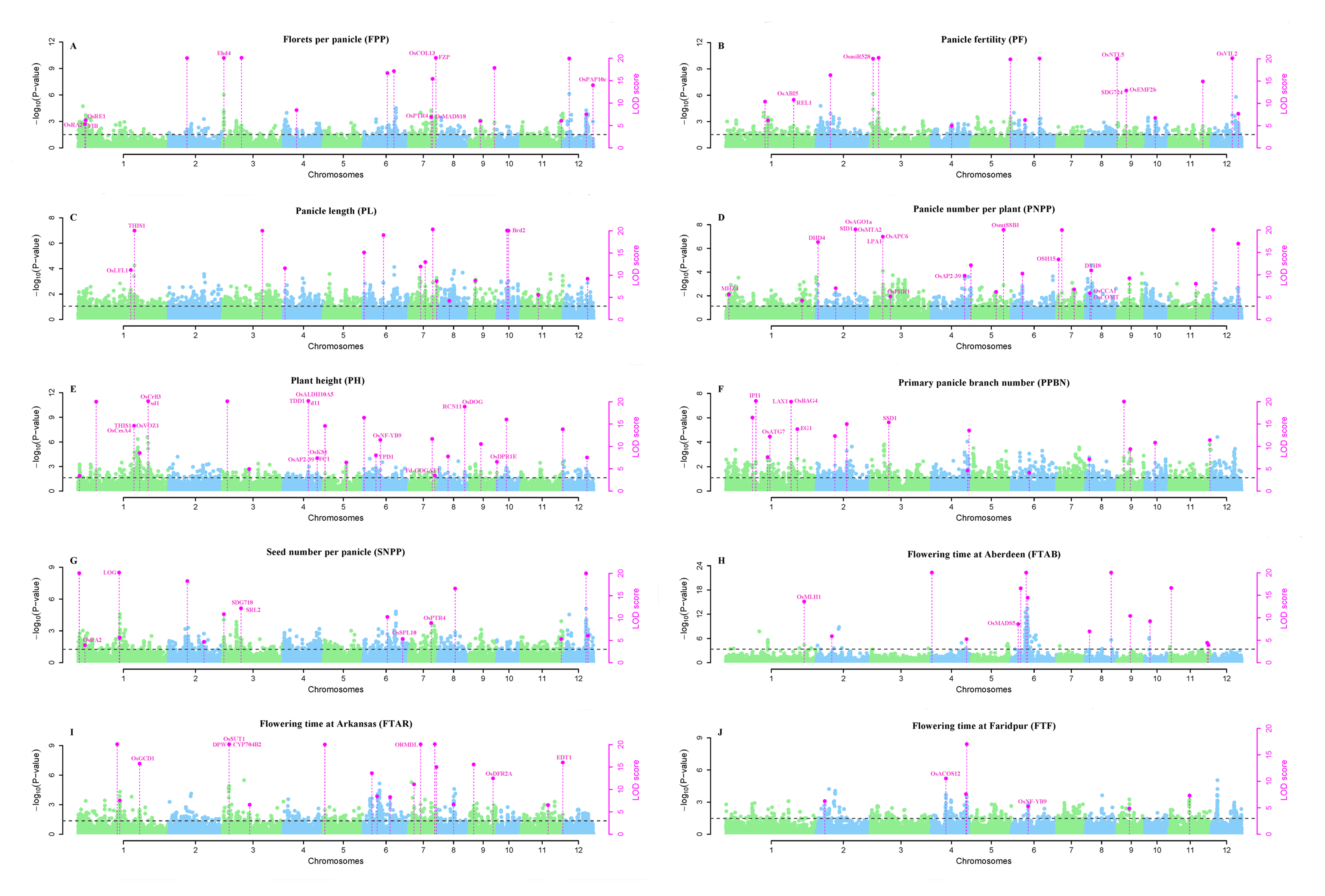

Supplement: Supplementary Figure 1 — Manhattan plots of the single-environment analysis for eight yield-related traits of rice. FTAB, FTAR, and FTF are the flowering time in three different environments. Pink text: known genes for the corresponding significant/suggested SNPs. [file Image_1.tif]

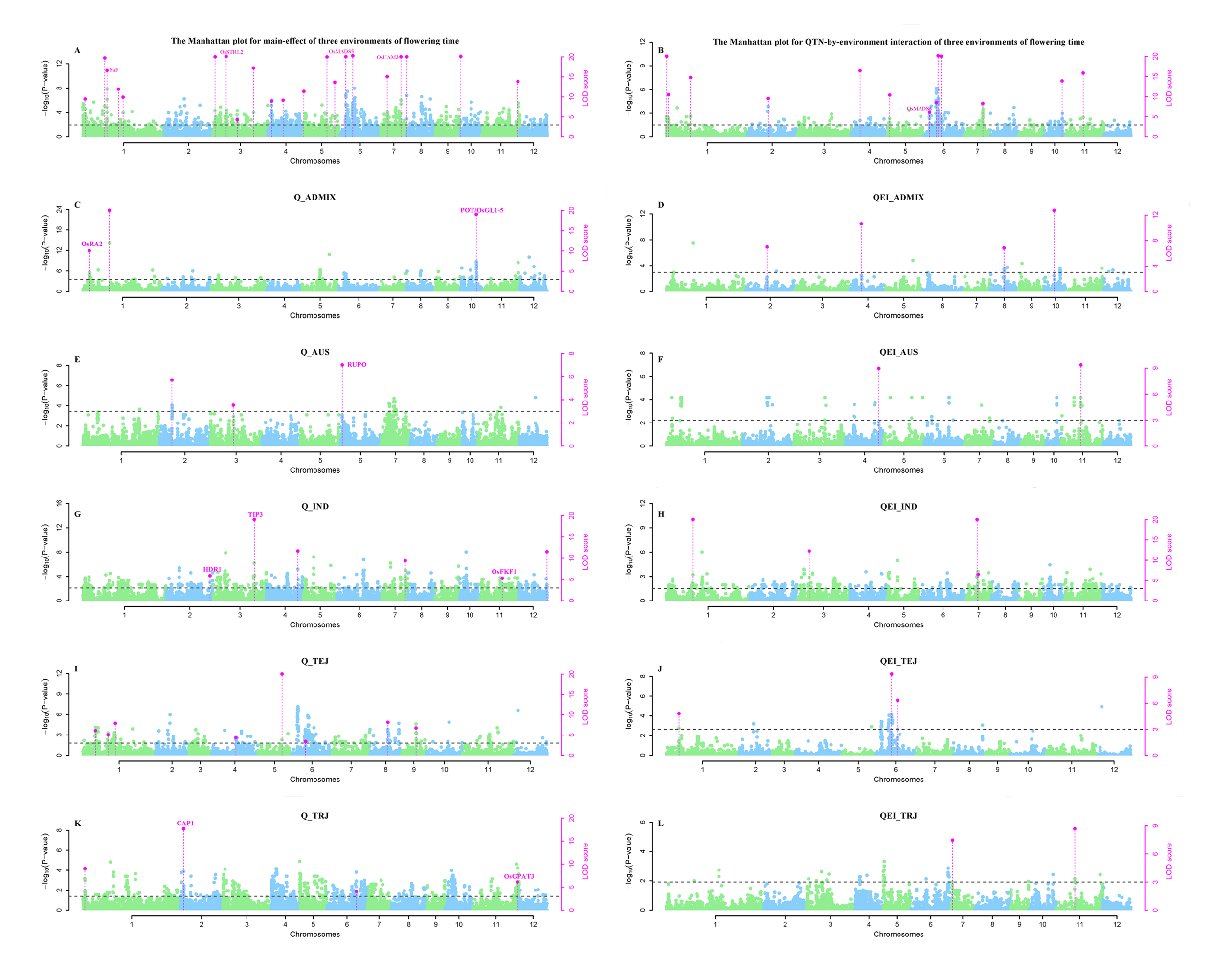

Supplement: Supplementary Figure 2 — Manhattan plots of the multi-environment analysis for the flowering time of rice. (A, B) Manhattan plots of QTNs and QTN-by-environment interactions for all populations. (C–L) Manhattan plots of QTNs and QTN-by-environment interactions for each subpopulation. Pink text: known genes for the corresponding significant/suggested SNPs. [file Image_2.tif]

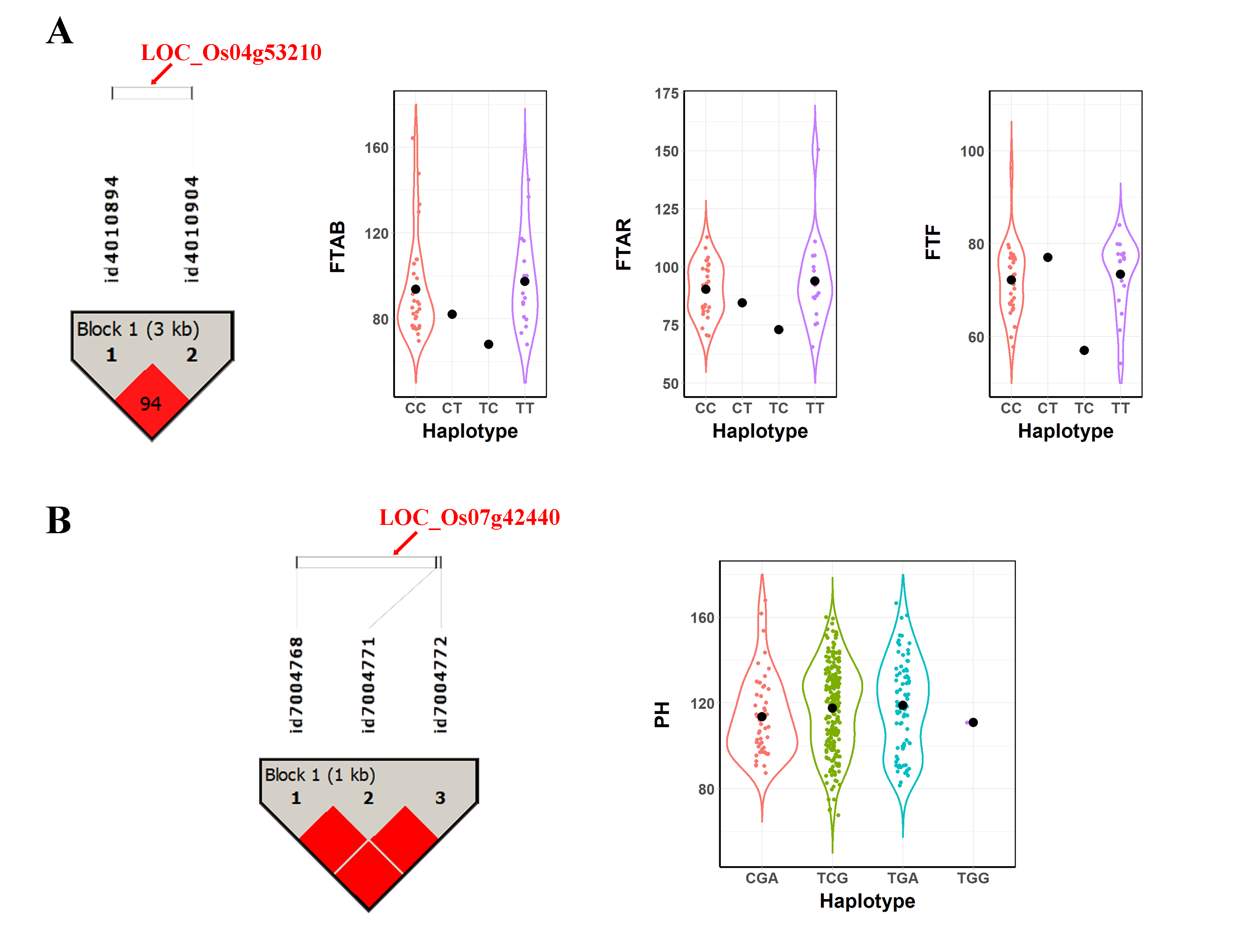

Supplement: Supplementary Figure 3 — Results of haplotype and phenotypic difference analysis for the candidate genes. (A) LOC_Os04g53210. (B) LOC_Os07g42440. [file Image_3.tif]
